# Supplementary material for: Phase-specific premotor inhibition modulates leech rhythmic motor output
Source: eLife. 2026 Jan 8;14:RP104921. doi: 10.7554/eLife.104921 (PMC12782552; doi:10.7554/eLife.104921)
Supplement: Supplementary file 1. — For each analyzed variable, the use of either a linear mixed model (LMM) or a generalized linear mixed model (GLMM) is specified, together with the structure of the random effects. In the case of GLMMs, the distribution and the corresponding link function are also specified. For each model, significance levels, p values, and ratios or estimated values are reported. [file elife-104921-supp1.docx]

| **Effect of NS depolarization on crawling features.** | | | | | |
| --- | --- | --- | --- | --- | --- |
| **DE-3** (n units = 26 ctrl, 19 depo / n leeches = 16 ctrl, 15 depo) | | | | | |
| Variable | Model, random effects | Significance of factor interactions | Pairwise simple contrasts | | |
|  |  |  | comparison | ratio | p value |
| FF (Hz) | LMM intercept - unit slope - epoch | <.0001 | ctrl depo / pre  post / pre post / depo  depo depo / pre post / pre post / depo | 1.04 1 0.96   1.62 0.96 0.59 | 0.6345 0.9815 0.4654   <.0001 0.3909 <.0001 |
| Burst duration (s) | GLMM - Gamma family, log link intercept - unit slope - epoch | 0.3077 | - | - | - |
| Period (s) | GLMM - Gamma family, log link intercept - unit | 0.2107 | - | - | - |
| Duty cycle | LMM intercept - unit slope - epoch | 0.8309 | - | - | - |
| **Effect of temporal drift con crawling features.** | | | | | |
| **DE-3** (n units = 26 ctrl, 19 depo / n leeches = 16 ctrl, 15 depo) | | | | | |
| Variable | Model, random effects | Significance of factor | Fixed effects | | |
|  |  |  | epoch | estimate | p value |
| Burst duration (s) | GLMM - Gamma family, log link intercept - unit | <.0001 | pre (intercept) depo post | 4.40 3.16 2.44 | <.0001 <.0001 <.0001 |
| Period (s) | GLMM - Gamma family, log link intercept - unit | <.0001 | pre (intercept) depo post | 15.45 11.45 9.47 | <.0001 <.0001 <.0001 |
| Duty cycle | LMM intercepto - unit | 0.1623 | - | - | - |
