# Supplementary material for: Phase-specific premotor inhibition modulates leech rhythmic motor output
Source: eLife. 2026 Jan 8;14:RP104921. doi: 10.7554/eLife.104921 (PMC12782552; doi:10.7554/eLife.104921)
Supplement: Supplementary file 2. — For each analyzed variable, the use of either a linear mixed model (LMM) or a generalized linear mixed model (GLMM) is specified, together with the structure of the random effects. In the case of GLMMs, the distribution and the corresponding link function are also specified. For each model, significance levels, p-values, and ratios or estimated values are reported. [file elife-104921-supp2.docx]

| **Effect of NS depolarization on motoneuron activity during *crawling*.** | | | | | |
| --- | --- | --- | --- | --- | --- |
| Variable | Model, random effects | Significance of factor interactions | Pairwise simple contrasts | | |
|  |  |  | comparison | ratio | p value |
| **DE-3** | | | | | |
| (n units = 26 ctrl, 20 depo / n cycles = 110 ctrl, 99 depo / n leeches = 16 ctrl, 16 depo) | | | | | |
| Max bFF (Hz) | LMM intercept - unit slope - treatment and epoch | <.0001 | ctrl depo / pre  post / pre post / depo  depo depo / pre post / pre post / depo | 1.04 1 0.96   1.59 0.89 0.56 | 0.5512 0.9995 0.5406   <.0001 0.0066 <.0001 |
| Relative HW | LMM intercept - unit slope - treatment and epoch | 0.1596 | - | - | - |
| **In-Phase** | | | | | |
| (n units = 34 ctrl, 19 depo / n cycles = 149 ctrl, 93 depo / n experiments = 17 ctrl, 11 depo / n leeches = 13 ctrl, 8 depo) | | | | | |
| Max bFF (Hz) | GLMM - t_family, identity link intercept - experiment/ unit | <.0001 | ctrl depo / pre post / pre post / depo  depo depo / pre post / pre post / depo | 1.01 0.92 0.91   1.52 0.86 0.56 | 0.9628 0.0046 0.0014   <.0001 0.0005 <.0001 |
| Relative HW | GLMM - t_family, identity link intercept - experiment/ unit | 0.0149 | ctrl depo / pre post / pre post / depo  depo depo / pre post / pre post / depo | 0.93 0.78 0.84   0.99 0.60 0.61 | 0.1788 <.0001 0.0007   0.9840 <.0001 <.0001 |
| **In-Phase-Early-Onset** | | | | | |
| (n units = 15 ctrl, 8 depo / n cycles = 66 ctrl, 45 depo / n experiments = 12 ctrl, 5 depo / n leeches = 11 ctrl, 5 depo) | | | | | |
| Max bFF (Hz) | LMM intercept - experiment/ unit | 0.0891 | - | - | - |
| Relative HW | GLMM - t_family, identity link intercept - experiment/ unit | 0.1730 | - | - | - |
| **Anti-Phase** | | | | | |
| (n units = 18 ctrl, 7 depo / n cycles = 78 ctrl, 34 depo / n experiments = 15 ctrl, 7 depo / n leeches = 12 ctrl, 7 depo) | | | | | |
| Max bFF (Hz) | GLMM - t_family, identity link intercept - experiment/ unit | 0.0015 | ctrl depo / pre post / pre post / depo  depo depo / pre post / pre post / depo | 0.91 0.82 0.90   1.10 0.94 0.85 | 0.0104 <.0001 0.0180   0.0505 0.4132 0.0025 |
| Relative HW | GLMM - t_family, identity link intercept - experiment/ unit | 0.9791 | - | - | - |
